# Embryo development until blastocyst stage with and without renewal of single medium on day 3

**DOI:** 10.5935/1518-0557.20180012

**Published:** 2018

**Authors:** Fernando Peña, Rocío Dávalos, Adolfo Rechkemmer, Alvaro Ascenzo, Mauricio Gonzales

**Affiliations:** 1 Laboratorio de Reproducción Asistida, Clínica Miraflores, Lima, Peru; 2 Laboratorio de Biotecnología Animal, Facultad de Ciencias, Universidad Ricardo Palma, Lima, Peru

**Keywords:** Embryo culture, single medium, uninterrupted culture, with and without culture renewal

## Abstract

**Objective:**

This study aimed to assess the effects of using a single culture medium not renewed on day 3 on the development of the embryo up to the blastocyst stage.

**Methods:**

The study was carried out in the Assisted Reproduction Laboratory of Clínica Miraflores in the period ranging from April to December of 2016. The study included 589 human embryos obtained from 82 couples submitted to IVF/ICSI with donor oocytes. The couples were randomly divided into two groups: group 1 (medium renewed on day 3) and group 2 (medium not renewed on day 3).

**Results:**

Significant differences in pregnancy rates, implantation rates, and blastocyst formation were not observed.

**Conclusion:**

No statistically significant difference was found between the groups with respect to the analyzed parameters. However, a non-significant trend was observed in favor of the group without medium renewal for clinical pregnancy and positive pregnancy rates (*β*-hCG).

## INTRODUCTION

Selecting the best embryo to transfer is a crucial step in *in vitro* fertilization (IVF) cycles. Before reaching this point, the selected embryos go through a series of events that might compromise their development and viability. One of the most remarkable is the days spent in culture medium environment.

Avoiding multiple gestation pregnancies is one of the most complex challenges faced in assisted reproductive technology therapies, which makes it necessary to extend the culture to the blastocyst stage to improve the odds of selecting good embryos, since early embryo development is inefficient and only a few zygotes eventually reach the blastocyst stage. In other words, embryos reaching the blastocyst stage undergo spontaneous selection in culture medium (Sepúlveda *et al.*, 2011; Gardner & Lane, 1998; Blake *et al*., 2007). Evidence suggests that higher pregnancy rates are achieved when embryos are transferred in the blastocyst stage rather than in the cleavage stage (Milki *et al.*, 2000).

The dynamic nature of early embryo development and the changes in the in vivo environment led to the formulation of sequential culture medium approaches designed to support embryos from the zygote to the blastocyst stage (Macklon *et al.*, 2002). Sequential culture medium approaches are based on the use of two culture media consecutively to support embryo development. The first contains pyruvate to provide the energy needed in the early stages of embryo development and cleavage, while the second contains glucose to meet the energy demands of blastocyst formation (Hardy *et al*., 1989).

Single culture medium approaches provide embryos with all nutrients needed for them to grow. Single culture medium has produced superior outcomes when compared to sequential culture medium, not only in terms of improved pregnancy rates but also in reference to the quality of the blastocysts obtained (Sepúlveda *et al.*, 2009; Biggers & Racowsky, 2002; Macklon *et al.*, 2002).

Comparisons between the two approaches have found that single culture medium yielded better outcomes in assisted reproductive technology therapies in the form of improved pregnancy rates and blastocyst formation (Vermilyea *et al.*, 2012). The option of not renewing the culture medium on day 3 has been proposed to avoid the manipulation of embryos in culture, but significant differences have not been found yet (Costa-Borges *et al.*, 2016; Rambhia & Desai, 2014). Therefore, the present study aimed to assess the effect of growing embryos to the blastocyst stage without renewing the culture medium on day 3.

## MATERIALS AND METHODS

The study was carried out in the Laboratory of Assisted Reproduction of Clínica Miraflores in the period ranging from April to December of 2016. The study included 589 human embryos obtained from 82 couples submitted to IVF/ICSI with donor oocytes. The couples were randomly divided into two groups: group 1 (medium renewed on day 3) and group 2 (medium not renewed on day 3).

### Ovarian stimulation and oocyte retrieval

For ovarian stimulation a dose of 150 to 300 IU/day of FSH and/or HMG was used during the first two to five days of the cycle based on patient age, body mass index, and response to previous stimulation. Dosage was adjusted according to ultrasound examination performed in intervals of two to three days. The patients were given GnRH antagonists and stimulation continued until the main follicles reached a mean diameter of 18mm. To trigger ovulation, human chorionic gonadotropin (hCG) or a GnRH agonist was administered; ovarian puncture was performed 36 hours later. Each patient received a mean of 8.4 donor oocytes. Severe male factor infertility was an exclusion criterion.

### Insemination and embryo culture

After ovarian puncture the oocytes were cultured in Global Total medium (LifeGlobal^®^) at 37°C, 5.5% CO_2_ and 5.0% O_2_ for 4.5 hours. Then the oocytes were inseminated by conventional IVF or intracytoplasmic sperm injection (ICSI). The oocytes inseminated by ICSI were denuded from cumulus cells by pipetting into Hyaluronidase 80 IU/ml (Fertipro^®^) and washed in Global Total medium with HEPES (LifeGlobal^®^). The metaphase II oocytes were injected with spermatozoa previously immobilized in PVP (Fertipro ^®^). After ICSI the oocytes were cultured in Global Total medium (LifeGlobal^®^) covered with 10ml of mineral oil (Fertipro^®^) for 5 to 6 days until transfer or vitrification. The oocytes inseminated by conventional IVF were kept in Global Total medium for fertilization (LifeGlobal^®^) at a concentration of 90,000 to 100,000 sperm per milliliter until the time of fertilization evaluation. Fertilization was evaluated approximately 18 hours after insemination. All cases were evaluated on day 3. Cases requiring medium renewal on day 3 were transferred to their new plates previously prepared with Global Total medium covered with mineral oil, while the cases without medium renewal were returned to the incubator after embryo assessment on day 3.

### Embryo assessment and selection for transfer

Embryo assessment was performed on day 3 including the following morphological parameters: number of blastomeres, degree of fragmentation, and symmetry between blastomeres. The blastocysts were assessed based on the ASEBIR classification system, which includes internal cell mass, trophectoderm, and degree of expansion of the blastocele. The blastocysts considered to be of good quality were selected for transfer, while the remaining ones that did not show signs of arrest or were of poorer quality were vitrified. All transfers occurred on day 5 of development with embryos in the blastocyst stage.

### Endometrial preparation for recipients

Patients with ovarian function were treated with intramuscular Leuprolide Acetate 3.75 mg on day 21 of the cycle preceding the IVF treatment. On the third day of the cycle, oral estradiol valerate 2 mg was administered for 5 days, followed by 4 mg daily for 5 days, and then 6mg daily as maintenance therapy. Menstruation was induced in patients without ovarian function through hormone replacement therapy; estradiol valerate was administered on day 3 of the treatment as described above.

To achieve luteal support, 1000mg/day of micronized Progesterone was administered vaginally to all recipients on the day of fertilization of the donor oocytes.

### Statistical analysis

The chi-square test was used to compare proportions, while the Student's t-test was used to compare quantities following a normal distribution. Statistical significance was ascribed to differences with a *p*-value<0.05. The Statistical Package for the Social Sciences 24.0 (SPSS Inc.) was used in data analysis and interpretation.

## RESULTS

The 689 metaphase II oocytes used in this study yielded 589 fertilized oocytes (Table 1). The oocytes had been divided into two groups, one featuring oocytes cultured in medium renewed on day 3 and another with oocytes cultured in single medium without renewal. Significant differences were not found in fertilization (83.02% vs. 87.60%), blastocyst formation (59.47% vs. 58.77%) or implantation (49.32% vs. 50.00%) rates.

**Table 1:** Embryo cultures with and without renewal

Clinical results	Total (%)	With renewal (%)	Without renewal (%)	*p-value*
nº of patients	82	38	44	-
nº of mature oocytes	689	318	371	0.901
Fertilization rate (%)	589 (85.49)	264 (83.02)	325 (87.60)	0.256
Blastocyst development (%)	348 (59.08)	157 (59.47)	191 (58.77)	0.269
nº of transferred embryos	159	73	86	0.725
Positive *β*-hCG (%)	54 (65.85)	24 (63.16)	30 (68.18)	0.632
Clinical pregnancy rate (%)	51 (62.2)	22 (57.89)	29 (65.91)	0.455
Implantation rate (%)	79 (49.69)	36 (49.32)	43 (50.00)	0.330

One hundred and fifty-nine blastocysts were transferred in the two groups combined, resulting in a positive pregnancy rate (*β*-hCG) of 63.16% vs. 68.18% for the groups with and without culture medium renewal, respectively. Clinical pregnancy and positive pregnancy rates (*β*-hCG) were slightly more favorable in the group without medium renewal (57.89% vs. 65.91%), but the difference was not statistically significant.

## DISCUSSION

The purpose of this study was to verify whether embryo culture in single medium without renewal on day 3 offered practical and physiological advantages over culture in single medium with renewal (Costa-Borges *et al.*, 2016; Biggers & Summers, 2008). IVF laboratories performing these procedures must comply with strict requirements to control the conditions to which embryo cultures are exposed, including temperature variations, light, and O_2_ and CO_2_ pressure (Cohen *et al.*, 1997). It is also important to control for substances that might affect the development of the embryos, such as dust particles, VOC, and disinfectants.

Some authors have not found significant differences in pregnancy or implantation rates when comparing cultures with and without medium renewal (Costa-Borges *et al.*, 2016; Rambhia & Desai, 2014). Similarly, our results did not reveal statistically significant differences when the two groups were compared, although increases of 8% in clinical pregnancy and 5% in positive pregnancy rates were observed (*β*-hCG).

Many authors have agreed that uninterrupted cultures produce more blastocysts for transfer or vitrification (Reed *et al.*, 2009; Vermilyea *et al.*, 2012). Even authors unable to find significant differences in pregnancy rates between the two methods (with or without medium renewal) have agreed that the blastocyst formation rate from uninterrupted cultures in single medium is significantly higher than that of embryo cultures with medium renewal on day 3 (Rambhia & Desai, 2014). Our results did not evince such pattern. The number of blastocysts available was not different from cultures with medium renewal, showing that single medium culture without medium renewal did not decrease the quality of the culture procedure, although it improved other parameters.

The application of this method might not only improve pregnancy rates as shown in previous studies, but also prevent the introduction of contaminants into the culture. Furthermore, it might decrease the stress caused to embryos by pipetting and variations in temperature and pH (Reed *et al.*, 2009). Avoiding embryo manipulation is a top priority in *in vitro* cultures to mitigate possible epigenetic risks (Velez de la Calle *et al.*, 2013).

The design of this study would have been improved if a cohort of sibling oocytes had been used. This measure would have allowed comparisons not only between patients, but also between embryos in the same cohort, removing the bias generated by circumstances occurring in the laboratory during the investigation.

## CONCLUSIONS

The results obtained in this study did not reveal statistically significant differences between cultures with and without medium renewal for the assessed parameters. However, a non-significant trend favoring cultures without medium renewal was observed in clinical pregnancy and positive pregnancy rates (*β*-hCG).
